# Field Trials Reveal Ecotype-Specific Responses to Mycorrhizal Inoculation in Rice

**DOI:** 10.1371/journal.pone.0167014

**Published:** 2016-12-01

**Authors:** Abdala Gamby Diedhiou, Fatou Kine Mbaye, Daouda Mbodj, Mathieu Ndigue Faye, Sarah Pignoly, Ibrahima Ndoye, Koffi Djaman, Souleymane Gaye, Aboubacry Kane, Laurent Laplaze, Baboucarr Manneh, Antony Champion

**Affiliations:** 1 Université Cheikh Anta Diop (UCAD), Faculté des Sciences et Techniques, Département de Biologie Végétale, Dakar-Fann, Sénégal; 2 Laboratoire Mixte International Adaptation des Plantes et microorganismes associés aux Stress Environnementaux, Centre de Recherche de Bel Air, Dakar, Sénégal; 3 Laboratoire Commun de Microbiologie IRD/ISRA/UCAD, Centre de Recherche de Bel Air, Dakar, Sénégal; 4 Institut de Recherche pour le Développement (IRD), UMR DIADE, Equipe CERES, Montpellier, France; 5 Africa Rice Center (AfricaRice), Saint-Louis, Senegal; Estacion Experimental del Zaidin, SPAIN

## Abstract

The overuse of agricultural chemicals such as fertilizer and pesticides aimed at increasing crop yield results in environmental damage, particularly in the Sahelian zone where soils are fragile. Crop inoculation with beneficial soil microbes appears as a good alternative for reducing agricultural chemical needs, especially for small farmers. This, however, requires selecting optimal combinations of crop varieties and beneficial microbes tested in field conditions. In this study, we investigated the response of rice plants to inoculation with arbuscular mycorrhizal fungi (AMF) and plant growth promoting bacteria (PGPB) under screenhouse and field conditions in two consecutive seasons in Senegal. Evaluation of single and mixed inoculations with AMF and PGPB was conducted on rice (*Oryza sativa*) variety Sahel 202, on sterile soil under screenhouse conditions. We observed that inoculated plants, especially plants treated with AMF, grew taller, matured earlier and had higher grain yield than the non-inoculated plants. Mixed inoculation trials with two AMF strains were then conducted under irrigated field conditions with four *O*. *sativa* varieties, two *O*. *glaberrima* varieties and two interspecific NERICA varieties, belonging to 3 ecotypes (upland, irrigated, and rainfed lowland). We observed that the upland varieties had the best responses to inoculation, especially with regards to grain yield, harvest index and spikelet fertility. These results show the potential of using AMF to improve rice production with less chemical fertilizers and present new opportunities for the genetic improvement in rice to transfer the ability of forming beneficial rice-microbe associations into high yielding varieties in order to increase further rice yield potentials.

## Introduction

Rice (*Oryza saliva* L.) is one of the oldest staple crops in the world [1], and the main source of calories for more than half of humanity [2]. To meet global needs, a 40% increase in production of rice must be achieved in the next 20 years on limited and increasingly degraded arable lands and in an unstable global climate context [3–4]. Sub-Saharan Africa is largely dependent on rice import for its food security. Incentive policies were set up to increase local rice production with three objectives: creation and dissemination of high-yielding varieties, development of irrigation facilities and availability of inorganic fertilizers. In countries such as Senegal, this has led to increased crop yields and quality [5–6]. However, the yields are still low [7] and the prohibitive cost and environmental problems caused by chemical inputs [8–10] support the search for new sustainable strategies to promote soil fertility and improve rice production. These approaches include the application of organic fertilizers, the use of nitrogen-fixing green manure (*Azolla* sp., fallow legumes) and of beneficial rhizospheric microorganisms such as arbuscular mycorrhizal fungi (AMF) and plant growth promoting bacteria (PGPB) and the selection of root systems for improved water and nutrient acquisition [4].

The arbuscular mycorrhizal (AM) symbiosis is a mutual relationship between plant roots and soil fungi belonging to the Glomeromycota [11]. In exchange for an allocation of plant carbon, the fungal partner provides water and minerals it collects in the soil to the plant [12]. The fungus creates a complex network of hyphae specialized in the absorption of minerals such as phosphorus and nitrogen in the soil and chimeric organs called arbuscules in the plant root cell that allow the exchange of resources with the plant host [13]. Through this symbiosis, plant species are able to exploit soil niches previously inaccessible [14]. In addition, the fungus improves the adaptability and resilience of its host to occasional or prolonged abiotic and biotic stress conditions [15–16]. Numerous studies have shown that mycorrhizal symbiosis induced significant changes in plant host architecture [17], and harvest index in rice in lab conditions [18]. However, AM symbiosis occurrence and plant responsiveness depend on environmental conditions, and specific plant and fungus combinations [19–22]. Exploiting the AM symbiosis potential for rice thus requires the selection of suitable combination of cultivar, fungus and agriculture practice. Moreover, co-inoculation with other beneficial microorganisms such as PGPB could positively improve AM symbiosis formation and functioning. Positive effects of PGPB on soil fertility and crop yield are well documented [23–24], and include mobilization of mineral or organic bound nutrients [25–27] and biological nitrogen fixation [28–29]. In rice, the impact of simple inoculations with AM fungi, diazotroph bacteria such as rhizobia and actinomycetes has been reported [29–30], but little is known about co-inoculation of consortia of such different plant growth promoting microorganisms [31]. Moreover, these studies have been performed in pot experiments but rice response to inoculation in field conditions is poorly documented.

The aims of this study were (1) to assess the responsiveness of rice to different combinations of four inoculants (AM fungi: *Glomus aggregatum* and *Rhizophagus irregulare* and PGPB: *Bradyrhizobium* sp. ORS 278 and *Leifsonia* sp. ORS 3454), (2) to identify the most effective inoculants combination, and (3) to test this combination in field experiments on eight varieties of rice.

## Materials and Methods

### Soil and plant materials

Pot trials were carried out twice (July 2013 and July 2015) with the same treatments in a screenhouse. The treatments consisted of non-inoculated and inoculated plants of rice (*O*. *sativa)* variety Sahel 202 with two AMF and two strains of PGPB applied as simple and mixed inoculants.

The soil used was collected from rice fields in Djibelor (12°33' N, 16°19' W) in the Casamance region of Senegal. The rice fields are privately owned lands, and permission to collect soil samples was obtained from the owners. The collected soil contained 1.32% total C, 0.08% total N, and 710 ppm total P. It was sieved with 2 mm sieves, sterilized twice at 180°C for 2 h and placed into plastic pots (1000 g of soil per pot).

Seeds of *O*. *sativa* Sahel 202, were surface-sterilized in 8.4% NaClO for 1 min and then 30 s in 90% ethanol, and washed 5 times in sterile, distilled water after each treatment. For pre-germination, seeds were put on moist filter paper under sterile conditions and placed in the dark (at 25°C). One day-old seedlings were planted in plastic pots (3 grains per pot) containing the culture substrate. Seedlings were thinned to one plant per pot two weeks after planting.

### Fungal materials and AMF inoculum production

The AM fungi used in this study were *Glomus aggregatum* Schenck & Smith (DAOM 227128, National Mycological Herbarium, Ottawa, Canada) and *Rhizophagus irregularis* Walker & Schüßler (previously called *Glomus intraradices* DAOM 197198; [32]). They were propagated as pure cultures in a greenhouse using a mycotrophic plant (*Zea mays*) and sterilized (2 x 2 h at 180°C) soil from Sangalkam (Senegal) consisting of 88.8% sand, 5.8% silt, 5.4% clay, 0.6% organic matter, 0.3% total C, 0.02% total N, 333.5 ppm total K, and 41.4 ppm total P. After 3 months, maize roots and culture substrate were collected to assess spore density [33] and the length of root colonized by AMF [34]. The colonized maize roots were cut into ~1 cm fragments and thoroughly homogenized to the culture substrate to constitute the AMF inoculum. For each AMF strain, the inoculum consisted of a mixture of sandy soil, spores (~500 / 100g of soil) and mycorrhizal root fragments (~70% of colonization rate).

### Bacterial materials and PGPB inoculum production

The bacterial strains used in this study were ORS278 and ORS3454 identified as *Bradyrhizobium* sp. [35] and *Leifsonia* sp. (99% of 16S rDNA sequence similarity with *Leifsonia shenshuensis*; Diégane Diouf, personal communication), respectively. The photosynthetic *Bradyrhizobium* sp. strain ORS278, was isolated from the aquatic legume, *Aeschynomene sensitiva*, in the Casamance region of Senegal [35]. The *Leifsonia* sp. strain ORS3454 was collected from pond water harboring wild rice plants (*Oryza barthii*) at Ndiaffate (Kaolack, Senegal; Diégane Diouf, personal communication). The plant growth promoting potential of the bacterial strain ORS278 has been reported [36–37], while that of the strain ORS3454 is under investigation. For each bacterial strain, a liquid culture (36°C, 180 rpm) in 500 ml of yeast extract-mannitol (YM) medium [38] was prepared from 1 ml of pre-culture from a single colony. In early stationary phase (2 and 6 days of culture for ORS3454 and ORS278 respectively) liquid cultures were centrifugated at 8000 rpm for 10 min. Bacterial pellets were washed 3 times (8000 rpm, 10 min) and suspended with sterile physiological water (8770 ppm NaCl, 270 ppm KH_2_PO_4_, 710 ppm Na_2_PO_4_) for plant inoculation.

### Seedling inoculations and experimental design

The inoculation experiment was made as follows: (a) simple and mixed inoculations with PGPB, abbreviated as ORS278, ORS3454, and ORS278 + ORS3454; (b) simple and mixed inoculations with AMF, *R*. *irregularis* (Ri), *G*. *aggregatum* (Ga), and Ga + Ri; (c) 9 mixed inoculations with AMF and PGPB; and (d) a control represented by the non-inoculated plants. Ten replicates were performed for each of the sixteen treatments arranged randomly in a screenhouse.

At planting time, 20 g of AMF inoculum were placed at a depth of ~4 cm in the center of pots and thoroughly mixed with sterilized soil. For the treatment with both AMF inoculants (Ga + Ri), 10 g of each were put in each pot. The treatments without AMF received an equivalent amount of sterilized inoculum (2 x 2 h at 180°C).

Inoculation with PGPB was performed 3 weeks after sowing, when rice plants reached the 4 leaves stage. At this stage, plants produce sufficient root exudates [39] to allow the development and maintenance of a rhizospheric bacteria population [40]. Before inoculation with PGPB, rice plants were exposed to water stress for 36 h to promote the absorption of bacterial inoculum in the rhizosphere. For each PGPB treatment, 10 ml of bacterial suspension (10^8^ CFU) were carefully instilled on seedling roots. The treatment with both PGPB inoculants (ORS3454 + ORS278) concomitantly received 5 ml suspension of each strain. The plants without PGPB inoculation received 10 ml of sterile physiological water. To avoid inoculum leaching, plant watering was resumed 18h later. A second inoculation with PGPB was performed 5 weeks after sowing to ensure the successful implementation of selected bacteria populations. In screenhouse experiments, rice plants were watered regularly with tap water to field capacity.

### Measurement of plant morphological and yield traits

Plant height and cross-sectional area of the stem base were measured every week for 3 weeks after sowing. Plant height was determined from the base of the main shoot to the tip of the longest leaf. Because rice plants have an approximate ellipsoidal stem base [41], the cross-sectional area was determined by measuring the diameters of long and short axis at the base of stem and applying the formula S = πDxDy / 4, where S is the cross-sectional area of the stem base, Dx and Dy are the diameters of long and short axis of the stem base, respectively. To reduce the bias related to heterogeneous seedling emergence, average increases of height and cross-sectional area of the stem base from the first date of measurement were considered.

The average of heading and maturity dates were determined for each treatment, and expressed in days after sowing (DAS). In this study, an experimental unit was considered to start heading if at least one panicle emerges from the leaf sheath. It reaches maturity when 80% of all of its spikelets are ripe. For each experimental unit, individual plants were harvested at maturity, and the panicles were weighed after air-drying in a room at 25°C to a constant weight.

### AMF colonization estimation

Roots were harvested and thoroughly washed with tap water. Large lateral roots which are more likely to form mycorrhizas [17] were collected, cleared in KOH (10% (w/v)) at 80°C for 30 min, and stained with trypan blue (0.05% (w/v) in 0.8% acid acetic solution) at 80°C for 35 min (adapted from Phillips and Hayman, [42]). Frequency of colonization and percentage of root length colonized by AMF were assessed for each treatment following the method used by Trouvelot, [34].

### Field experiments

Field trials were carried out in two consecutive years (September 2013 to January 2014 and September 2014 to January 2015), at the AfricaRice Sahel Station at Ndiaye (16°14' N, 16°14' W), with the permission from the AfricaRice Sahel Station Director. Treatments consisted of non-inoculated and inoculated plants of 8 rice varieties: 4 *O*. *sativa* (Sahel 108, Sahel 202, IR 64 and WAB 56–104), two *O*. *glaberrima* (TOG 5681 and CG 14) and two interspecific varieties (NERICA 4 and NERICA–L-19). The ecotype and some agronomic traits of the different varieties are presented in Table 1.

**Table 1:** Ecotype and some agronomic traits of rice cultivars used in the present study.

Variety	Species / Parents	Ecotype	Days to 50% maturity	1000GWT (g)	Potential yield (t/ha)
Sahel 108	*O*. *sativa indica*	Irrigated (Irr)	105–120	23–24	10
Sahel 202	*O*. *sativa indica*	Irrigated (Irr)	115	27	11
IR 64	*O*. *sativa indica*	Irrigated (Irr)	118	26	4–5
WAB 56–104	*O*. *sativa japonica*	Upland (Upl)	105	31	4
TOG 5681	*O*. *glaberrima*	Rainfed lowland (Rll)	nd	nd	nd
CG 14	*O*. *glaberrima*	Upland (Upl)	nd	nd	nd
NERICA 4	*O*. *sativa japonica x O*. *glaberrima* (WAB 56–104 / CG 14 // 2*WAB 56–104)	Upland (Upl)	95–100	29	5
NERICA–L-19	*O*. *glaberrima x O*. *sativa indica* (TOG 5681/3*IR 64)	Rainfed lowland (Rll)	140	23	8

1000GWT: 1000 grain weight.

For each rice variety, inoculation of seedlings was performed in nursery as follows: 3 pre-germinated seeds were planted in each pot (4cm x 4cm x 4cm) of multi-pot plates filled with a mixture of 20 g of Ga + Ri inoculum and 20 g of sterilized Sangalkam soil. Twelve days after planting, non-inoculated (planted in multi-pot plates with the sterilized mixture) and inoculated seedlings were sampled to check the establishment of arbuscular mycorrhizae in each rice variety. AMF structures were observed in roots of all inoculated seedlings, while AMF colonization was not observed in the non-inoculated plants. 13 day-old mycorrhized and non-mycorrhized seedlings were transferred in field plots, according to a split-plot design with 3 replications: the block with inoculated seedlings and that with non-inoculated seedlings were considered as main plots, and the 8 rice varieties were assigned to subplots. Thus, 24 (8 varieties x 3 replications) experimental units of 0.48 m^2^ each were set up in both blocks with inoculated and non-inoculated seedlings. In each experimental unit, 12 seedlings were transplanted and maintained in irrigated conditions with 20 x 20 cm spacing and one plant per hill.

Both blocks with inoculated and non-inoculated seedlings were treated with fertilizers as recommended: 130 kg/ha of DAP, 100kg/ha of KCl NPK (23kg of N– 60 kg of P2O5–60 kg of K2O) and 10 kg/ha of zinc were applied two weeks after transplanting. 276 kg/ha of urea (46-0-0 NPK) was applied in three split applications: 40% at early tillering (2 weeks after transplanting), 40% at panicle initiation (4 to 6 weeks after transplanting) and 20% at booting stage (9 weeks after transplanting).

Four rice hills in the center of each subplot were harvested at maturity and the following agronomic traits were assessed: plants height, number of tillers, grain yield and 1000 grain weight (both expressed at 14% moisture), aboveground biomass (at 14% moisture), harvest index (HI, defined as the ratio of grain yield to aboveground biomass), spikelet fertility (defined as the ratio of the number of filled spikelets to the total number of spikelets), and grain filling duration (GFD, defined as the period between flowering and physiological maturity). The days to 50% heading (defined as the time when 50% of the rice plants had exserted their panicles) and to 80% maturity (when 80% of grains had lost green color) were also recorded.

### Data analysis

In the screenhouse experiments, root length and frequency of colonization, heading, maturity and panicle weight were analyzed by a one-way analysis of variance (ANOVA) with inoculum (control, simple and mixed inoculants) as factor. In the field experiments, a three-way ANOVA was performed to analyze for effects of inoculation (control and AMF), year of trial (1^st^ year and 2^nd^ year) and rice variety (each of the 8 varieties tested) or rice ecotype (upland, irrigated and rainfed lowland) on the 10 agronomic traits; while differences between two sample means were determined by a Student’s t-test. Prior to analysis, data were ln (x + 10) transformed to meet assumptions of normality, and significant differences in means were determined at *P*<0.05 using the XLSAT™ software package (2010 version, Addinsoft).

For each rice variety, the mycorrhizal inoculation effect (MIE, indicating the effect of introduced AMF inoculum compared with the inherent field inoculums), was calculated for each agronomic trait as follows: MIE = (mean value of inoculated plants–mean value of non-inoculated plants) / mean value of inoculated plants. MIE varies between -1 and 1. For morphological traits, a positive MIE indicates that the plants benefited from introduced AMF inoculum, while a negative MIE means that the costs for the introduced AMF are higher than mycorrhizal benefit. To examine ecotype-specific responses to AMF inoculation, a Non-metric multidimensional scaling (NMDS) based on a Bray-Curtis similarity measure was performed using the MIE values for agronomic traits that showed significant AMF inoculation x ecotype interaction in ANOVA. Similarity percentages (SIMPER) analysis on the basis of Bray-Curtis dissimilarities was than conducted to identify the agronomic traits that contributed most to the differences recorded between rice ecotypes in terms of response to inoculation with AMF, by using the PAST software package (version 3.12).

## Results

### AMF inoculation increases rice growth and hastens maturity

**Fig 1:**
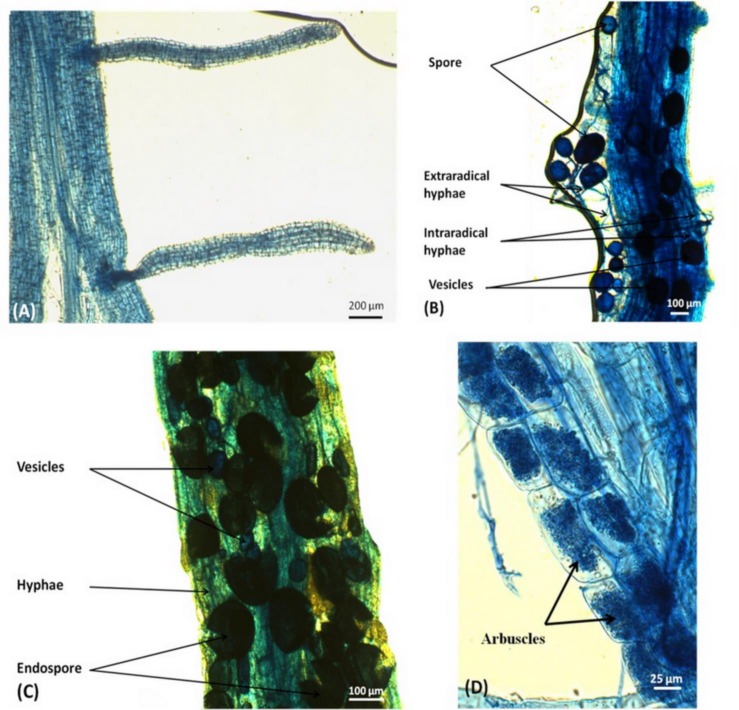
Roots of *O*. *sativa* var. Sahel 202 with and without AMF structures. Roots free of AMF structures (A); root fragment colonized by *G*. *aggregatum*, with extraradical spores (B); root fragment colonized by *R*. *irregularis* presenting typical endospores (C); and root fragment colonized by *G*. *aggregatum*, with arbuscules (D).

We first assessed the responsiveness of the rice Sahel 202 variety to different AM fungi and PGPB combination in pot experiments. In two independent trials, no AMF colonization was observed in the roots of non-inoculated plants, whereas typical AM structures such as arbuscules, hyphae and vesicles were observed within the roots of plants inoculated with one or the two AMF strains (*G*. *aggregatum* and *R*. *irregularis*) alone or in combination with PGPR strains (*Bradyrhizobium* sp. ORS278 and *Leifsonia* sp. ORS3454). Spores and typical *Rhizophagus* endospores were also observed (Fig 1). Combination of the fungal strains or co-inoculations with PGPB did not increase AMF colonization (S1 Table).

Independent trials revealed that microbial inoculations had a positive effect on rice growth in pot (S1 and S2 Figs). Moreover, simple and mixed inoculants including at least one AMF significantly hastened heading and maturity of *O*. *sativa* Sahel 202 plants and significantly increased panicle weight. By contrast, simple and mixed bacterial inoculants had no significant effects on these traits (Table 2). Hence, in pot experiments, inoculation of *O*. *sativa* Sahel 202 variety with a combination of AMF strains increased both rice plant height and vigor and reduced the duration of the growth cycle.

**Table 2:** *O*. *sativa* var. Sahel 202 heading and maturity dates and panicle weight for the 1^st^ and 2^nd^ year trials.

	Heading (DAS)		Maturity (DAS)		Panicle weight (mg)	
Traitment	1^st^ year	2^nd^ year	1^st^ year	2^nd^ year	1^st^ year	2^nd^ year
Control	126.83 a	130.00 ab	151.00 ab	157.00 a	699.10 d	688.57 de
ORS278	123.50 a	125.13 abc	152.33 a	153.00 abc	1006.20 cd	608.89 e
ORS3454	115.89 ab	130.00 ab	143.00 abc	155.40 ab	1134.80 bcd	656.25 e
ORS278 + ORS3454	119.25 a	121.63 bcd	147.17 abc	153.00 abc	1129.90 bcd	922.22 cde
Ri	95.78 c	128.00 abc	136.25 cd	153.00 abc	1854.90 abc	904.44 cde
Ri + ORS278	96.00 de	113.40 de	144.22 abc	140.70 de	1647.30 abc	1077.00 abc
Ri + ORS3454	101.00 cd	112.00 ef	154.40 a	137.90 ef	1733.50 abc	918.00 cde
Ri + ORS278 + ORS3454	106.33 bc	109.20 ef	137.62 bcd	135.10 ef	1727.80 abc	1035.00 bc
Ga	101.60 cd	105.00 f	138.22 bcd	133.78 f	1872.70 abc	1325.00 ab
Ga + ORS278	103.11 cd	105.00 f	136.33 cd	140.70 de	1658.60 abc	1386.00 a
Ga + ORS3454	98.20 cd	109.20 ef	136.00 cd	140.70 de	2271.60 a	1092.00 abc
Ga + ORS278 + ORS3454	92.17 de	106.40 ef	128.83 de	137.20 ef	2134.10 a	1334.00 ab
Ga + Ri	89.00 e	112.00 ef	122.00 e	140.00 def	1942.20 ab	917.00 cde
Ga + Ri + ORS278	97.44 cde	104.30 f	139.00 bcd	133.78 f	1709.40 abc	867.00 cde
Ga + Ri + ORS3454	98.22 cde	120.56 cd	131.00 de	146.22 cd	2026.30 a	1091.11 abc
Ga + Ri + ORS278 + ORS3454	101.29 cd	110.60 ef	130.20 de	138.60 ef	2155.50 a	1021.25 bcd

In each column, means followed by the same letter are not significantly different at *P*≤0.05. DAS: days after sowing.

### AMF inoculation impacts agronomic traits of rice varieties in irrigated field conditions

**Fig 2:**
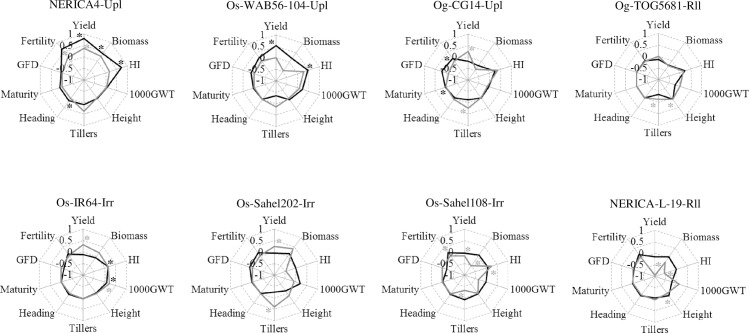
Mycorrhizal inoculation effect (MIE) for the 10 agronomic traits of each rice variety in both first (black line) and second (grey line) year trials. Abbreviations associated to the variety names indicate the rice species (Os: *O*. *sativa*; Og: *O*. *glaberrima*) and ecotype (Upl: Upland, Irr: Irrigated, Rll: Rainfed lowland). HI: harvest index; 1000GWT: 1000 grain weight; GFD: grain filling duration. Stars indicate that the means of inoculated plants and non-inoculated plants were significantly different (*P*<0.05).

We next tested the impact of AMF inoculation on 8 varieties of rice corresponding to different species (four *O*. *sativa*, two *O*. *glaberrima* and two interspecific NERICA) and ecotypes (upland, irrigated and rainfed lowland) in field conditions. The analysis of grain yield revealed a significant interaction between inoculation with AMF and rice variety (*P* < 0.000), which itself depended on year of trial (*P* = 0.001 for AMF x variety x year interaction, S2 Table). In the first year trial, only two upland rice varieties, NERICA 4 and *O*. *sativa* WAB56-104, showed significant increase in grain yield when inoculated with AMF (Table 3 and S3 Table), with strong positive MIE (0.80 and 0.52, respectively; Fig 2). In the second year trial, significant differences in grain yield between the inoculated and non-inoculated plants were obtained in 6 rice varieties (Table 3 and S3 Table), with positive MIE in two upland rice varieties (NERICA 4 and *O*. *glaberrima* CG14), and two irrigated rice varieties (*O*. *sativa* IR64 and Sahel 202), and negative MIE in the rainfed lowland variety NERICA-L-19 and the irrigated variety Sahel 108 (Fig 2).

**Table 3:** Agronomic traits of inoculated and non-inoculated plants of the 8 rice varieties cultivated under irrigated filed conditions.

	Treat	Yield (Kg/ha)	Biomass (Kg/ha)	HI (%)	1000GWT (g)	Height (cm)	Tillers numb	Heading (DAS)	Maturity (DAS)	GFD (Days)	Fertility (%)
NERICA4	AM-1	**3730 a**	**8648 a**	**52 a**	28	102	165	**75 a**	107	32	80
	NM-1	**0734 b**	**5063 b**	**15 b**	26	102	152	**66 b**	097	31	27
WAB56-104	AM-1	**2438 a**	5411	**52 a**	32	87	123	73	112	39	73
	NM-1	**1169 b**	4354	**28 b**	25	85	167	73	107	34	54
CG14	AM-1	3646	10252	45	29	102	336	73	**99 a**	26	**76 a**
	NM-1	4355	12646	34	30	106	381	75	**96 b**	20	**64 b**
IR64	AM-1	4834	12657	**46 a**	**25 a**	78	346	81	112	31	64
	NM-1	5340	13667	**39 b**	**23 b**	79	334	78	112	34	56
Sahel202	AM-1	4938	17792	34	27	78	302	84	110	26	60
	NM-1	5180	15250	34	21	88	378	84	108	23	49
Sahel108	AM-1	4188	11486	45	19	78	286	79	98	18	74
	NM-1	4378	10584	42	19	77	265	76	98	22	60
NERICA-L-19	AM-1	5625	14709	46	23	87	279	91	114	23	74
	NM-1	6440	14084	47	28	82	294	87	111	24	61
TOG5681	AM-1	4334	09623	54	26	77	303	69	96	27	66
	NM-1	4905	11855	42	32	71	409	69	96	27	65
NERICA4	AM-2	**4631 a**	10906	44	24	100	325	74	105	31	**89 a**
	NM-2	**2862 b**	08079	36	23	103	204	74	105	31	**71 b**
WAB56-104	AM-2	3283	07810	42	26	84	317	73	106	33	80
	NM-2	3290	11378	30	24	88	275	73	106	33	74
CG14	AM-2	**5103 a**	12719	40	19	099	**540 a**	74	102	28	87
	NM-2	**3904 b**	18104	25	21	100	**410 b**	70	101	31	86
IR64	AM-2	**6104 a**	14263	43	**24 a**	80	483	98	130	31	93
	NM-2	**4236 b**	11525	37	**19 b**	81	463	98	130	31	88
Sahel202	AM-2	**5234 a**	25274	21	21	94	**500 a**	91	125	33	91
	NM-2	**3953 b**	15028	31	22	84	**304 b**	91	125	33	87
Sahel108	AM-2	**7692 b**	**13756 b**	**57 a**	18	80	**438 b**	75	106	31	**92 a**
	NM-2	**8959 a**	**20868 a**	**43 b**	25	79	**579 a**	73	104	31	**89 b**
NERICA-L-19	AM-2	**2363 b**	21246	**11 b**	30	**85 b**	575	92	124	32	85
	NM-2	**4609 a**	26141	**18 a**	26	**91 a**	556	92	124	32	79
TOG5681	AM-2	3993	10769	38	26	**81 a**	**435 b**	74	98	24	86
	NM-2	4016	13543	33	26	**74 b**	**490 a**	74	98	24	89

Treat: treatment; AM: inoculated with AMF; NM: control (without AMF); Number associated to AM and NM indicates the year of trial (1: first year and 2: second year); HI: harvest index; 1000GWT: 1000 grain weight; GFD: grain filling duration; numb: number; DAS: days after sowing. In each column, means followed by different letters are significantly different (*P*≤0.05).

For the aboveground biomass, there was a significant interaction between inoculation with AMF and rice variety (*P* = 0.002), while this interaction was independent of year of trial (S2 Table). NERICA 4 was the only rice variety whose aboveground biomass was significantly increased when inoculated with AMF in the first year trial (Table 3 and S3 Table). In contrast, the aboveground biomass was significantly decreased by the inoculation with AMF in Sahel 108 (Table 3 and S3 Table), with strong negative MIE (-0.52) in the second year trial (Fig 2).

The analysis of the harvest index revealed a significant interaction between inoculation with AMF and rice variety (*P* = 0.000), which itself depended on year of trial (*P* = 0.048 for AMF x variety x year interaction, S2 Table). Therefore, significant differences in harvest index between the inoculated and non-inoculated plants were obtained in 3 varieties (NERICA 4, WAB56-104, and IR64) and 2 varieties (Sahel 108 and NERICA-L-19) in the first and second year trial, respectively (Table 3 and S3 Table). These varieties, except NERICA-L-19, displayed positive MIE ranging from 0.72 in NERICA 4 to 0.15 in IR64 (Fig 2).

For tillers number, ANOVA revealed a significant interaction between inoculation with AMF and rice variety (*P* = 0.032), which itself depended on year of trial (*P* = 0.011 for AMF x variety x year interaction, S2 Table). Hence, significant positive effects of inoculation with AMF were recorded in 2 varieties (Sahel 202 and CG14), whereas 2 other varieties (Sahel 108 and TOG5681) displayed significant negative effects of inoculation with AMF in the second year trial (Fig 2, Table 3).

ANOVA for spikelet fertility revealed that the effect of inoculation with AMF was significantly dependent on rice variety (*P* = 0.000 for AMF x variety interaction, S2 Table). Of the 8 rice varieties, *O*. *glaberrima* CG14 (first year trial), and NERICA 4 and Sahel108 (second year trial) showed significant increase in spikelet fertility when inoculated with AMF (Table 3 and S3 Table), with the highest MIE (0.657) recorded in NERICA 4 (Fig 2).

### Mycorrhizal response profiling of the 8 rice varieties according to their ecotypes

**Fig 3:**
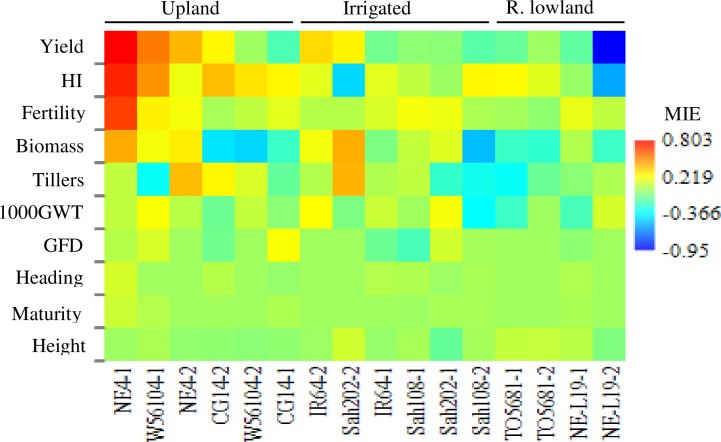
Matrix plot depicting the response to inoculation with AMF (MIE) of rice at variety and ecotype levels. HI: harvest index; 1000GWT: 1000 grain weight; GFD: grain filling duration. Numbers associated to the variety names (NE: NERICA; W: WAB; Sah: Sahel; TO: TOG) indicate the year of trial (1: first year and 2: second year).

ANOVA revealed that the effect of inoculation with AMF was significantly dependent on rice ecotype for grain yield (*P* = 0.002 for AMF x ecotype interaction), harvest index (*P* = 0.005 for AMF x ecotype interaction), and spikelet fertility (*P* = 0.037 for AMF x ecotype interaction). In addition, the AMF x ecotype interaction for the different agronomic traits was independent of year of trial (S4 Table). Indeed, only upland varieties showed significant positive effects of inoculation with AMF for these 3 agronomic traits in both first and second year trials (S5 Table). Fig 3 showed how MIE for each agronomic trait varied among the rice ecotypes, with MIE values ranging from strong positive in upland varieties to strong negative in rainfed lowland varieties particularly for yield, harvest index and spikelet fertility.

**Fig 4:**
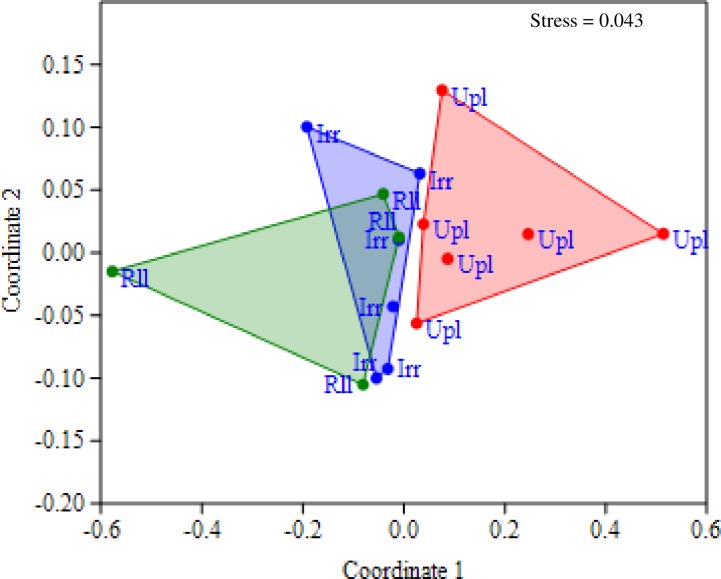
NMDS representation of the rice ecotypes based on the Bray-Curtis similarity measure of their response to inoculation with AMF (MIE) for yield, harvest index and spikelet fertility. Abbreviations Upl, Irr and Rll indicate the upland, irrigated and rainfed lowland rice ecotypes, respectively. To reduce the stress value, a three-dimensional ordination space was chosen of which two coordinates are shown.

A two-dimensional NMDS ordination plot comparing the ecotype responses to inoculation with AMF for yield, harvest index and spikelet fertility in both first and second year trials is shown in Fig 4. The NMDS plot which presented small stress value (0.043), clearly separated the upland varieties from the irrigated and lowland varieties with a partial overlap for these latter ecotypes. Similarity percentages (SIMPER) analysis on the basis of Bray-Curtis dissimilarities revealed 0.899, 0.627 and 0.556 of average dissimilarity for upland vs rainfed lowland, upland vs irrigated, and irrigated vs rainfed lowland, respectively. SIMPER also indicated that yield and harvest index were responsible for more than 80% of the differences recorded between ecotypes in terms of response to inoculation with AMF (Table 4).

**Table 4:** Contribution of agronomic traits to the differences recorded in response to inoculation with AMF (MIE) of rice ecotypes in both first and second year trials, revealed by Similarity percentage (SIMPER).

	Upland *vs* Rainfed lowland			Upland *vs* Irrigated			Irrigated *vs* Rainfed lowland		
Agro. traits	Aver. dissim.	Contrib. %	Cumul. %	Aver. dissim.	Contrib. %	Cumul. %	Aver. dissim.	Contrib. %	Cumul. %
YLD	0.453	50.34	50.34	0.260	41.47	41.47	0.279	50.18	50.18
HI	0.308	34.27	84.61	0.250	39.87	81.34	0.209	37.59	87.77
FRT	0.138	15.39	100	0.117	18.66	100	0.068	12.23	100
Overall	0.899			0.627			0.556		

Agro.: agronomic; Aver. dissim.: average dissimilarity; Contrib.: contribution; Cumul.: cumulative; YLD: yield; HI: harvest index; FRT: fertility.

## Discussion

In this study, we analyzed the impact of inoculation with beneficial soil microorganisms on rice growth and yield in controlled and field conditions over two years. Our results revealed a positive response of the irrigated rice variety Sahel 202 to inoculation with simple and mixed microbial inoculants in pot experiments. Indeed, significant improvement of growth and panicle weight as well as earliness of heading and maturity, were noticed in plants inoculated with one or the two AMF strains alone or in combination with PGPB strains. On the other hand, significant effects of simple and mixed PGPB inoculants were only observed on plant height. These results suggest that plant response to inoculation is related to the composition and diversity of microbial communities [43]. This hypothesis is partially supported by the finding that the beneficial effects of single AMF inoculation on plant growth can result from different mechanisms [44], reflecting some functional diversity among AM fungi. For instance, the capabilities for nutrient (especially phosphate) acquisition through enzyme activities and/or extra-radical mycelia that act as an extension of the host root system differ substantially among AMF [11]. As nutrients in the soil have a patchy distribution [45–46], co-occurrence of different strains in the same root system can lead to a "functional complementarity" in the fungal exploration of nutrient niches surrounding the roots [46–47]. Accordingly, although there was no significant difference in frequency of colonization and percentage of root length colonized by *G*. *aggregatum* and *R*. *irregularis* alone and in combination, plants inoculated with a combination of both AMF showed earlier heading and maturity compared to that inoculated with only one AMF. This indicates that the effect of plant inoculation with AMF on some rice agronomic traits is not directly linked to the degree of root colonization by AMF.

One of the most interesting phenotypes we observed in response to AMF inoculation in pots experiments was a shortening of the time to flowering and maturity. For most plant species, in the absence of phenological events, flowering occurs after the plant reaches a fit vegetative development [48–49]. Hence, improving nutrition by AMF would have caused the shortening of the vegetative phase as reported in tomato and *Abutilon theophrasti* [50–51]. Shortening the development cycle without adverse effects on yield would save inputs, limit the exposure of crops to climate instabilities and give more flexibility to the timing of cropping calendars.

We therefore tested whether this was translatable to field conditions with 8 rice varieties corresponding to different species (four *O*. *sativa*, two *O*. *glaberrima* and two interspecific NERICA) and ecotypes (upland, irrigated and rainfed lowland). Significant impacts of inoculation with AMF on agronomic traits were observed in all rice varieties. All analyzed agronomic traits, except grain filling duration, were significantly increased in at least one rice variety. Our results clearly show that rice response to AMF inoculation under irrigated field conditions depends on varieties. Importantly, the effects of AMF inoculation on *O*. *sativa* Sahel 202 were very different in pot and field experiments thus demonstrating the need to analyze the impact of AMF inoculants in field conditions. This discrepancy might be due to the impact of anoxic conditions due to flooding in field on the survival and function of AM symbiosis.

Interestingly, we observed that plant response to AMF inoculation is in large part related to the plant ecotype. Upland varieties tended to respond positively to AMF inoculation in contrast to rainfed lowland and irrigated varieties in both trials. It has been documented that the interaction between AMF and its host plant can range functionally along a parasitism mutualism continuum depending on soil resources and plant species, and in particular on root morphology and architecture [52–53]. Indeed, mycorrhizal dependency is often high in plants with thick and poorly branched roots and low in plants with thin and highly branched roots [11, 52]. In our study, root morphology and architecture of the different rice varieties were not analyzed. However, it has been suggested that *O*. *sativa* Indica types (Group 1, mostly lowland) have thin, highly branched roots, while tropical Japonica types (Group 6, which include upland Asian and temperate cultivars) have thick, less-branched long roots [54]. As a consequence, tropical Japonica types would display higher mycorrhizal responsiveness than the Indica types. Accordingly, upland rice varieties including the Japonica WAB 56–104 displayed strong positive MIE for most of the analyzed agronomic traits, whereas Indica types (IR64, Sahel 202 and Sahel 108; irrigated) displayed moderate positive or negative MIE. Furthermore, the interspecific variety NERICA 4 (upland) has *O*. *sativa japonica* and *O*. *glaberrima* parents (Table 1) and displayed strong positive MIE. On the other hand, the NERICA-L-19 variety (lowland) has *O*. *sativa indica* and *O*. *glaberrima* parents, showed strong negative MIE. This suggests that the differences observed in the mycorrhizal responsiveness of the 8 rice varieties cultivated under irrigated field conditions might be linked to root morphology and architecture regarding the ecotype, although other explanations may account for these features.

Altogether, the results of this study reveal ecotype-specific responses to AMF inoculation which could be an important tool to improve rice yields and resilience in Africa and in particular for upland rice production systems that have the greatest potential for growth. Future studies will focus on the identification of optimal inoculum combinations as well as rice genome regions that control the establishment of symbiotic associations between AMF and rice.

## Supporting Information

S1 Fig Height of non-inoculated and inoculated plants of *O*. *sativa* Sahel 202. A single microbial strain (AMF or PGPR, A), two strains (B), and 3 and 4 strains (C), were used in the 1^st^ year (A1, B1 and C1) and 2^nd^ year (A2, B2 and C2) trials. Ri: *Rhizophagus irregularis*; Ga: *Glomus aggregatum*; ORS 278: *Bradyrhizobium* sp. ORS 278; and ORS 3454: *Leifsonia* sp. ORS 3454.
(TIF)

S2 Fig Collar section growth curves of non-inoculated and inoculated plants of *O*. *sativa* Sahel 202. A single microbial strain (AMF or PGPR, D1 and D2), two strains (E1 and E2), and 3 and 4 strains (F1 and F2), were used in the 1^st^ year (D1, E1 and F1) and 2^nd^ year (D2, E2 and F2) trials. Ri: *Rhizophagus irregularis*; Ga: *Glomus aggregatum*; ORS 278: *Bradyrhizobium* sp. ORS 278; and ORS 3454: *Leifsonia* sp. ORS 3454.
(TIF)

S1 Table Root length and frequency of colonization of inoculated plants of *O*. *sativa* var. Sahel 202 for the 1^st^ and 2^nd^-year trials. In each column, means followed by the same letter are not significantly different at *P*≤0.05.
(PDF)

S2 Table ANOVA for the ln (x +10) transformed values of agronomic traits in rice plants at variety level. AMF inoculation (inoculated and non-inoculated), variety (each of the 8 varieties tested) and year (1^st^ and 2^nd^-year trial).
(PDF)

S3 Table Student’s t-test for the ln (x +10) transformed values of agronomic traits in rice plants at variety level. AM: inoculated with AMF and NM: non-inoculated. Abbreviations associated to the variety names indicate the rice ecotype (Upl: Upland, Irr: Irrigated, Rll: Rainfed lowland).
(PDF)

S4 Table ANOVA for the ln (x +10) transformed values of agronomic traits in rice plants at ecotype level. AMF inoculation (inoculated and non-inoculated), ecotype (upland, irrigated and rainfed lowland) and year (1^st^ and 2^nd^-year trial).
(PDF)

S5 Table Student’s t-test for the ln (x +10) transformed values of agronomic traits in rice plants at ecotype level. AM: inoculated with AMF and NM: non-inoculated.
(PDF)
