# Supplementary figures and images for: Field Trials Reveal Ecotype-Specific Responses to Mycorrhizal Inoculation in Rice
Source: PLoS One. 2016 Dec 1;11(12):e0167014. doi: 10.1371/journal.pone.0167014 (PMC5132163; doi:10.1371/journal.pone.0167014)

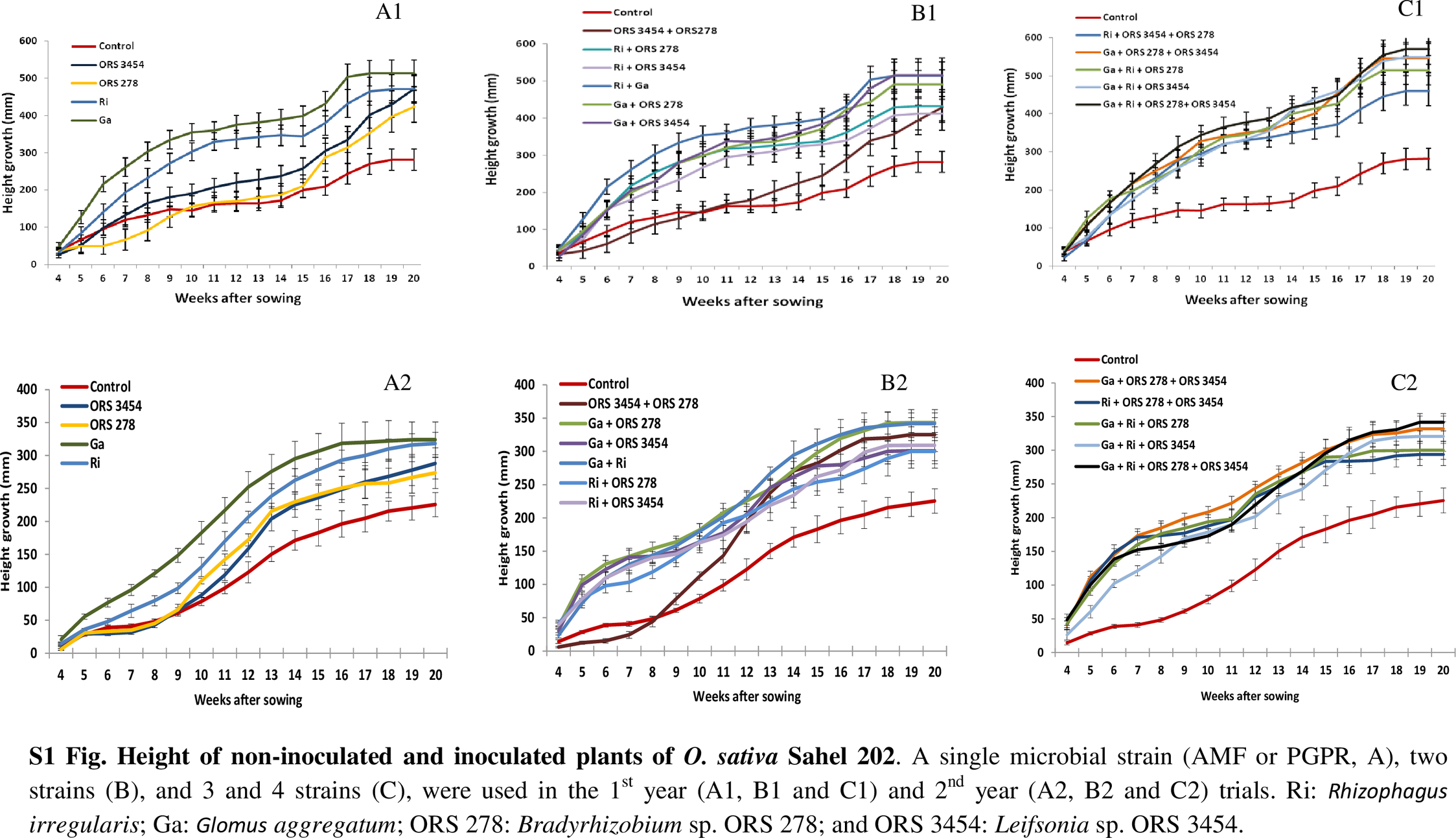

Supplement: S1 Fig — A single microbial strain (AMF or PGPR, A), two strains (B), and 3 and 4 strains (C), were used in the 1st year (A1, B1 and C1) and 2nd year (A2, B2 and C2) trials. Ri: Rhizophagus irregularis; Ga: Glomus aggregatum; ORS 278: Bradyrhizobium sp. ORS 278; and ORS 3454: Leifsonia sp. ORS 3454. (TIF) [file pone.0167014.s001.tif]

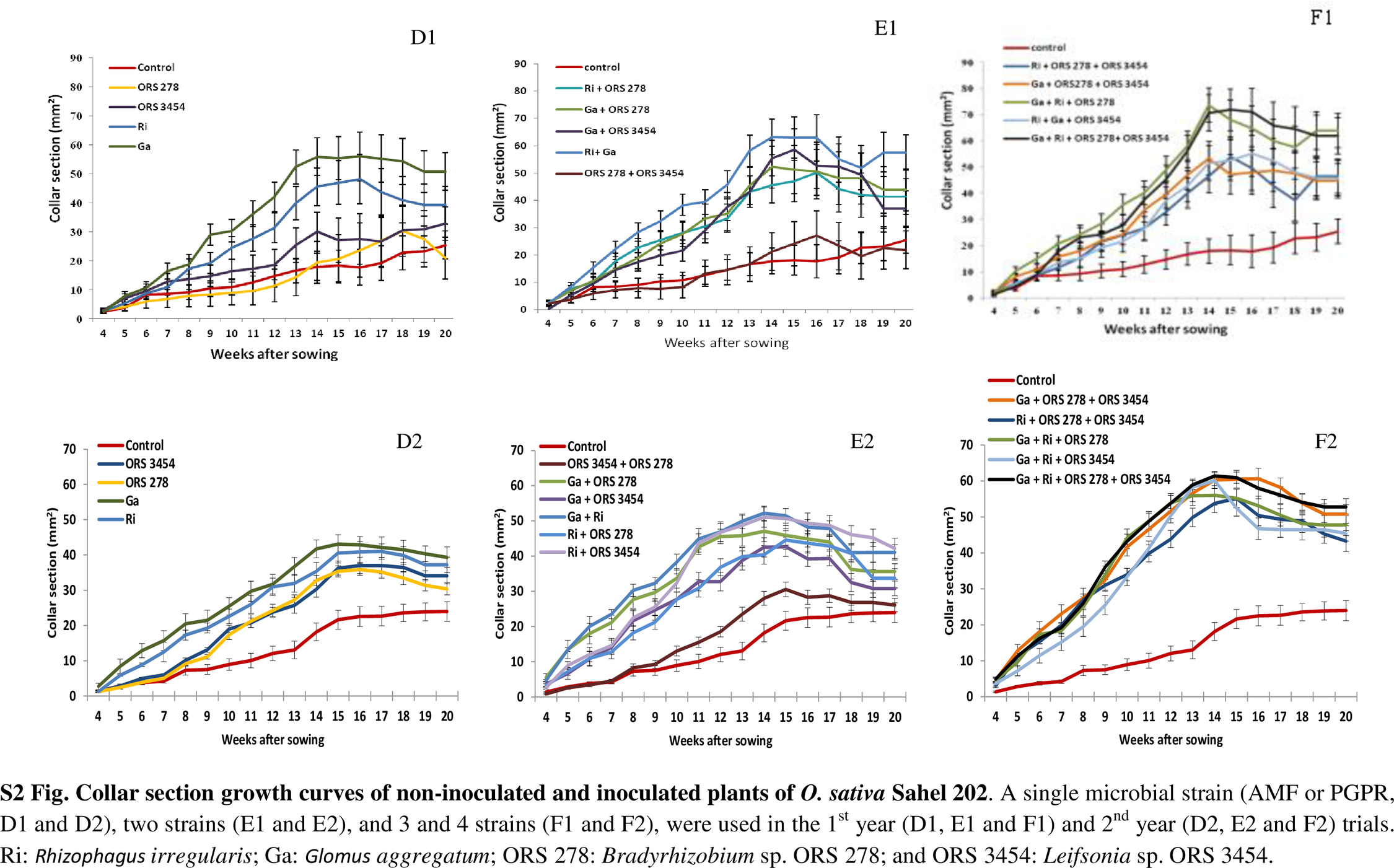

Supplement: S2 Fig — A single microbial strain (AMF or PGPR, D1 and D2), two strains (E1 and E2), and 3 and 4 strains (F1 and F2), were used in the 1st year (D1, E1 and F1) and 2nd year (D2, E2 and F2) trials. Ri: Rhizophagus irregularis; Ga: Glomus aggregatum; ORS 278: Bradyrhizobium sp. ORS 278; and ORS 3454: Leifsonia sp. ORS 3454. (TIF) [file pone.0167014.s002.tif]
