# Supplementary material for: Field Trials Reveal Ecotype-Specific Responses to Mycorrhizal Inoculation in Rice
Source: PLoS One. 2016 Dec 1;11(12):e0167014. doi: 10.1371/journal.pone.0167014 (PMC5132163; doi:10.1371/journal.pone.0167014)
Supplement: S1 Table — In each column, means followed by the same letter are not significantly different at P≤0.05. (PDF) [file pone.0167014.s003.pdf]

S1 Table. Root length and frequency of colonization of inoculated plants of *O. sativa* var. Sahel 202 for the 1st and 2nd-year trials. In each column, means followed by the same letter are not significantly different at $P \leq 0.05$.

Traitment	Root lenght colonization		Frequency of colonization	
	1st year	2nd year	1st year	2nd year
Ri	60.08 ab	38.70 d	98.19 a	92.00 cd
Ri + ORS278	52.54 b	38.70 d	94.14 ab	92.00 cd
Ri + ORS3454	56.60 ab	36.85 def	98.29 a	90.50 de
Ri + ORS278 + ORS3454	51.62 b	32.50 f	99.34 a	86.50 ef
Ga	49.33 b	55.41 a	93.82 ab	98.00 ab
Ga + ORS278	55.35 b	50.08 bc	98.60 a	97.00 abc
Ga + ORS3454	52.24 b	53.75 ab	98.64 a	98.50 a
Ga + ORS278 + ORS3454	57.65 ab	55.28 a	97.25 ab	96.50 abc
Ga + Ri	53.94 b	47.33 c	97.49 a	94.50 abcd
Ga + Ri + ORS278	61.56 ab	34.10 def	98.23 a	86.00 ef
Ga + Ri + ORS3454	50.24 b	33.70 ef	93.74 ab	85.00 f
Ga + Ri + ORS278 + ORS3454	71.58 a	37.60 de	99.40 a	93.00 bcd
