# Supplementary material for: Field Trials Reveal Ecotype-Specific Responses to Mycorrhizal Inoculation in Rice
Source: PLoS One. 2016 Dec 1;11(12):e0167014. doi: 10.1371/journal.pone.0167014 (PMC5132163; doi:10.1371/journal.pone.0167014)
Supplement: S2 Table — AMF inoculation (inoculated and non-inoculated), variety (each of the 8 varieties tested) and year (1st and 2nd-year trial). (PDF) [file pone.0167014.s004.pdf]

**S2 Table. ANOVA for the ln (x +10) transformed values of agronomic traits in rice plants at variety level**. AMF inoculation (inoculated and non-inoculated), variety (each of the 8 varieties tested) and year (1st and 2nd-year trial).

| Yield | | | | | |
|---|---|---|---|---|---|
| Source of variation | df | *SS* | *MS* | *F* | *P* |
| AMF inoculation (AMF n = 2) | 1 | 0.641 | 0.641 | 7.654 | 0.007 |
| Variety (Var n = 8) | 7 | 10.563 | 1.509 | 18.006 | < 0.0001 |
| Year (Yr n = 2) | 1 | 1.091 | 1.091 | 13.022 | 0.001 |
| AMF x Var | 7 | 5.028 | 0.718 | 8.571 | < 0.0001 |
| AMF x Yr | 1 | 0.192 | 0.192 | 2.289 | 0.135 |
| Var x Yr | 7 | 5.522 | 0.789 | 9.414 | < 0.0001 |
| AMF x Var x yr | 7 | 2.367 | 0.338 | 4.035 | 0.001 |
| Error | 64 | | | | |
| Biomass | | | | | |
| Source of variation | df | *SS* | *MS* | *F* | *P* |
| AMF inoculation (AMF n = 2) | 1 | 0.003 | 0.003 | 0.057 | 0.811 |
| Variety (Var n = 8) | 7 | 10.675 | 1.525 | 28.690 | < 0.0001 |
| Year (Yr n = 2) | 1 | 2.250 | 2.250 | 42.322 | < 0.0001 |
| AMF x Var | 7 | 1.397 | 0.200 | 3.754 | 0.002 |
| AMF x Yr | 1 | 0.082 | 0.082 | 1.549 | 0.218 |
| Var x Yr | 7 | 1.166 | 0.167 | 3.132 | 0.007 |
| AMF x Var x yr | 7 | 0.640 | 0.091 | 1.720 | 0.120 |
| Error | 64 | | | | |
| Harvest index (HI) | | | | | |
| Source of variation | df | *SS* | *MS* | *F* | *P* |
| AMF inoculation (AMF n = 2) | 1 | 0.767 | 0.767 | 19.626 | < 0.0001 |
| Variety (Var n = 8) | 7 | 1.706 | 0.244 | 6.237 | < 0.0001 |
| Year (Yr n = 2) | 1 | 0.535 | 0.535 | 13.696 | 0.000 |
| AMF x Var | 7 | 1.239 | 0.177 | 4.530 | 0.000 |
| AMF x Yr | 1 | 0.134 | 0.134 | 3.434 | 0.068 |
| Var x Yr | 7 | 2.244 | 0.321 | 8.207 | < 0.0001 |
| AMF x Var x yr | 7 | 0.596 | 0.085 | 2.178 | 0.048 |
| Error | 64 | | | | |
| 1000 grains weight (1000GWT) | | | | | |
| Source of variation | df | *SS* | *MS* | *F* | *P* |
| AMF inoculation (AMF n = 2) | 1 | 0.012 | 0.012 | 0.276 | 0.601 |
| Variety (Var n = 8) | 7 | 0.457 | 0.065 | 1.510 | 0.180 |
| Year (Yr n = 2) | 1 | 0.078 | 0.078 | 1.804 | 0.184 |
| AMF x Var | 7 | 0.211 | 0.030 | 0.699 | 0.673 |

| | | | | | |
|---|---|---|---|---|---|
| AMF x Yr | 1 | 0.010 | 0.010 | 0.231 | 0.633 |
| Var x Yr | 7 | 0.255 | 0.036 | 0.844 | 0.555 |
| AMF x Var x yr | 7 | 0.153 | 0.021 | 0.507 | 0.827 |
| Error | 64 | | | | |
| Height | | | | | |
| Source of variation | df | *SS* | *MS* | *F* | *P* |
| AMF inoculation (AMF n = 2) | 1 | 0.000 | 0.000 | 0.008 | 0.931 |
| Variety (Var n = 8) | 7 | 0.863 | 0.123 | 28.096 | < 0.0001 |
| Year (Yr n = 2) | 1 | 0.009 | 0.009 | 1.982 | 0.164 |
| AMF x Var | 7 | 0.021 | 0.003 | 0.693 | 0.678 |
| AMF x Yr | 1 | 0.000 | 0.000 | 0.013 | 0.909 |
| Var x Yr | 7 | 0.019 | 0.003 | 0.618 | 0.739 |
| AMF x Var x yr | 7 | 0.050 | 0.007 | 1.617 | 0.147 |
| Error | 64 | | | | |
| Tillers | | | | | |
| Source of variation | df | *SS* | *MS* | *F* | *P* |
| AMF inoculation (AMF n = 2) | 1 | 0.003 | 0.003 | 0.135 | 0.714 |
| Variety (Var n = 8) | 7 | 7.396 | 1.057 | 41.449 | < 0.0001 |
| Year (Yr n = 2) | 1 | 4.389 | 4.389 | 172.169 | < 0.0001 |
| AMF x Var | 7 | 0.424 | 0.061 | 2.374 | 0.032 |
| AMF x Yr | 1 | 0.300 | 0.300 | 11.782 | 0.001 |
| Var x Yr | 7 | 0.855 | 0.122 | 4.789 | 0.000 |
| AMF x Var x yr | 7 | 0.511 | 0.073 | 2.866 | 0.011 |
| Error | 64 | | | | |
| Heading | | | | | |
| Source of variation | df | *SS* | *MS* | *F* | *P* |
| AMF inoculation (AMF n = 2) | 1 | 0.006 | 0.006 | 4.996 | 0.029 |
| Variety (Var n = 8) | 7 | 0.687 | 0.098 | 79.494 | < 0.0001 |
| Year (Yr n = 2) | 1 | 0.045 | 0.045 | 36.369 | < 0.0001 |
| AMF x Var | 7 | 0.008 | 0.001 | 0.931 | 0.489 |
| AMF x Yr | 1 | 0.002 | 0.002 | 1.252 | 0.267 |
| Var x Yr | 7 | 0.112 | 0.016 | 12.947 | < 0.0001 |
| AMF x Var x yr | 7 | 0.014 | 0.002 | 1.605 | 0.150 |
| Error | 64 | | | | |
| Maturity | | | | | |
| Source of variation | df | *SS* | *MS* | *F* | *P* |
| AMF inoculation (AMF n = 2) | 1 | 0.005 | 0.005 | 8.496 | 0.005 |
| Variety (Var n = 8) | 7 | 0.496 | 0.071 | 124.127 | < 0.0001 |
| Year (Yr n = 2) | 1 | 0.089 | 0.089 | 155.334 | < 0.0001 |
| AMF x Var | 7 | 0.005 | 0.001 | 1.240 | 0.295 |
| AMF x Yr | 1 | 0.003 | 0.003 | 5.389 | 0.023 |
| Var x Yr | 7 | 0.063 | 0.009 | 15.861 | < 0.0001 |

| | | | | | |
|---|---|---|---|---|---|
| AMF x Var x yr | 7 | 0.006 | 0.001 | 1.624 | 0.145 |
| Error | 64 | | | | |

Grain filling duration (GFD)

| Source of variation | df | *SS* | *MS* | *F* | *P* |
|---|---|---|---|---|---|
| AMF inoculation (AMF n = 2) | 1 | 0.001 | 0.001 | 0.089 | 0.767 |
| Variety (Var n = 8) | 7 | 0.622 | 0.089 | 11.249 | < 0.0001 |
| Year (Yr n = 2) | 1 | 0.223 | 0.223 | 28.209 | < 0.0001 |
| AMF x Var | 7 | 0.038 | 0.005 | 0.693 | 0.677 |
| AMF x Yr | 1 | 0.005 | 0.005 | 0.585 | 0.447 |
| Var x Yr | 7 | 0.533 | 0.076 | 9.643 | < 0.0001 |
| AMF x Var x yr | 7 | 0.076 | 0.011 | 1.370 | 0.233 |
| Error | 64 | | | | |

Fertility

| Source of variation | df | *SS* | *MS* | *F* | *P* |
|---|---|---|---|---|---|
| AMF inoculation (AMF n = 2) | 1 | 0.597 | 0.597 | 25.018 | < 0.0001 |
| Variety (Var n = 8) | 7 | 0.511 | 0.073 | 3.060 | 0.008 |
| Year (Yr n = 2) | 1 | 2.183 | 2.183 | 91.508 | < 0.0001 |
| AMF x Var | 7 | 0.770 | 0.110 | 4.611 | 0.000 |
| AMF x Yr | 1 | 0.252 | 0.252 | 10.576 | 0.002 |
| Var x Yr | 7 | 0.355 | 0.051 | 2.125 | 0.053 |
| AMF x Var x yr | 7 | 0.358 | 0.051 | 2.143 | 0.051 |
| Error | 64 | | | | |
