# Supplementary material for: Field Trials Reveal Ecotype-Specific Responses to Mycorrhizal Inoculation in Rice
Source: PLoS One. 2016 Dec 1;11(12):e0167014. doi: 10.1371/journal.pone.0167014 (PMC5132163; doi:10.1371/journal.pone.0167014)
Supplement: S3 Table — AM: inoculated with AMF and NM: non-inoculated. Abbreviations associated to the variety names indicate the rice ecotype (Upl: Upland, Irr: Irrigated, Rll: Rainfed lowland). (PDF) [file pone.0167014.s005.pdf]

S3 Table. Student's t-test for the ln (x +10) transformed values of agronomic traits in rice plants at variety level. AM = inoculated with AMF and NM = non-inoculated. Abbreviations associated to the variety names indicate the rice ecotype (Upl = Upland, Irr = Irrigated, Rll = Rainfed lowland).

Yield								
	First year trial				Second year trial			
Variety	AM	NM	*t*-value	*P*-value	AM	NM	*t*-value	*P*-value
NERICA4-Upl	**8.214**	**6.241**	**2.915**	**0.043**	**8.437**	**7.937**	**2.810**	**0.048**
WAB56-104-Upl	**7.773**	**7.072**	**4.031**	**0.016**	8.098	8.102	-0.003	0.934
CG14-Upl	8.134	8.327	-0.531	0.623	**8.538**	**8.271**	**5.355**	**0.006**
IR64-Irr	8.485	8.546	-0.297	0.782	**8.717**	**8.350**	**5.212**	**0.006**
Sahel202-Irr	8.499	8.542	-0.309	0.773	**8.565**	**8.281**	**4.394**	**0.012**
Sahel108-Irr	8.340	8.380	-0.433	0.687	**8.949**	**9.101**	**-6.406**	**0.003**
NERICA-L-19-Rll	8.569	8.766	-0.750	0.495	**7.761**	**8.426**	**-4.310**	**0.013**
TOG5681-Rll	8.351	8.469	-0.484	0.654	8.292	8.297	-0.069	0.948
Biomass								
	First year trial				Second year trial			
Variety	AM	NM	*t*-value	*P*-value	AM	NM	*t*-value	*P*-value
NERICA4-Upl	**9.057**	**8.530**	**4.970**	**0.008**	9.285	8.984	1.829	0.141
WAB56-104-Upl	8.557	8.328	0.756	0.492	8.963	9.317	-2.247	0.088
CG14-Upl	9.233	9.420	-1.059	0.349	9.448	9.733	-0.984	0.381
IR64-Irr	9.445	9.497	-0.300	0.778	9.555	9.352	1.899	0.130
Sahel202-Irr	9.784	9.622	1.338	0.252	10.131	9.530	1.947	0.123
Sahel108-Irr	9.341	9.265	0.685	0.531	**9.525**	**9.945**	**-4.970**	**0.008**
NERICA-L-19-Rll	9.571	9.545	0.133	0.901	9.951	10.171	-1.901	0.130
TOG5681-Rll	9.157	9.379	-1.623	0.180	9.268	9.472	-0.831	0.453
Harvest index (HI)								
	First year trial				Second year trial			
Variety	AM	NM	*t*-value	*P*-value	AM	NM	*t*-value	*P*-value
NERICA4-Upl	**4.132**	**3.118**	**3.325**	**0.029**	3.969	3.810	1.097	0.334
WAB56-104-Upl	**4.107**	**3.628**	**3.899**	**0.018**	3.953	3.684	2.101	0.104
CG14-Upl	3.938	3.780	0.606	0.577	3.918	3.518	1.781	0.150
IR64-Irr	**4.031**	**3.897**	**3.517**	**0.025**	3.975	3.843	1.676	0.169
Sahel202-Irr	3.782	3.791	-0.158	0.882	3.431	3.668	-1.156	0.312
Sahel108-Irr	3.999	3.944	0.603	0.579	**4.193**	**3.970**	**3.291**	**0.030**
NERICA-L-19-Rll	4.007	4.034	-0.185	0.862	**3.054**	**3.314**	**-3.133**	**0.035**
TOG5681-Rll	4.157	3.933	1.884	0.133	3.866	3.720	0.692	0.527
1000 grains weight (1000GWT)								
	First year trial				Second year trial			
Variety	AM	NM	*t*-value	*P*-value	AM	NM	*t*-value	*P*-value
NERICA4-Upl	3.638	3.345	0.541	0.617	3.514	3.470	0.654	0.549
WAB56-104-Upl	3.715	3.548	1.104	0.331	3.572	3.520	0.505	0.640
CG14-Upl	3.663	3.696	-0.980	0.382	3.369	3.444	-1.020	0.365
IR64-Irr	**3.555**	**3.486**	**4.474**	**0.011**	**3.514**	**3.350**	**3.016**	**0.039**
Sahel202-Irr	3.581	3.444	0.942	0.399	3.419	3.477	-0.538	0.620

Sahel108-Irr	3.359	3.366	-0.070	0.948	3.344	3.526	-0.959	0.392
NERICA-L-19-Rll	3.494	3.624	-1.474	0.214	3.662	3.590	0.447	0.678
TOG5681-Rll	3.578	3.733	-1.523	0.203	3.569	3.573	-0.123	0.908

Height

	First year trial				Second year trial			
Variety	AM	NM	*t*-value	*P*-value	AM	NM	*t*-value	*P*-value
NERICA4-Upl	4.713	4.721	-0.162	0.880	4.697	4.725	-1.052	0.352
WAB56-104-Upl	4.576	4.550	0.330	0.758	4.542	4.584	-1.067	0.346
CG14-Upl	4.720	4.752	-0.534	0.621	4.690	4.722	-1.240	0.283
IR64-Irr	4.473	4.490	-0.292	0.785	4.502	4.510	-0.381	0.723
Sahel202-Irr	4.471	4.582	-1.519	0.203	4.644	4.536	2.104	0.103
Sahel108-Irr	4.481	4.466	0.702	0.521	4.497	4.488	0.385	0.720
NERICA-L-19-Rll	4.566	4.513	0.408	0.704	**4.551**	**4.613**	**-3.434**	**0.026**
TOG5681-Rll	4.465	4.394	1.832	0.141	**4.506**	**4.430**	**5.211**	**0.006**

Tillers

	First year trial				Second year trial			
Variety	AM	NM	*t*-value	*P*-value	AM	NM	*t*-value	*P*-value
NERICA4-Upl	5.162	5.082	0.774	0.482	5.782	5.359	2.125	0.101
WAB56-104-Upl	4.891	5.158	-1.932	0.126	5.763	5.639	0.622	0.568
CG14-Upl	5.830	5.957	-0.744	0.498	**6.303**	**6.040**	**3.018**	**0.039**
IR64-Irr	5.859	5.832	0.178	0.868	6.199	6.147	0.450	0.676
Sahel202-Irr	5.727	5.949	-1.308	0.261	**6.231**	**5.747**	**6.210**	**0.003**
Sahel108-Irr	5.683	5.613	0.698	0.524	**6.103**	**6.378**	**-7.715**	**0.002**
NERICA-L-19-Rll	5.640	5.716	-0.456	0.672	6.371	6.336	0.647	0.553
TOG5681-Rll	5.743	6.028	-2.655	0.057	**6.099**	**6.213**	**-3.769**	**0.020**

Heading

	First year trial				Second year trial			
Variety	AM	NM	*t*-value	*P*-value	AM	NM	*t*-value	*P*-value
NERICA4-Upl	**4.442**	**4.331**	**4.729**	**0.009**	4.430	4.430	0.000	1.000
WAB56-104-Upl	4.418	4.419	-0.017	0.987	4.423	4.423	0.000	1.000
CG14-Upl	4.422	4.446	-0.691	0.527	4.430	4.386	1.735	0.158
IR64-Irr	4.514	4.473	1.533	0.200	4.685	4.685	0.000	1.000
Sahel202-Irr	4.538	4.546	-0.164	0.878	4.618	4.618	0.000	1.000
Sahel108-Irr	4.492	4.458	1.285	0.268	4.442	4.423	0.677	0.535
NERICA-L-19-Rll	4.611	4.573	0.790	0.474	4.625	4.625	0.000	1.000
TOG5681-Rll	4.369	4.369	-0.015	0.989	4.435	4.435	0.000	1.000

Maturity

	First year trial				Second year trial			
Variety	AM	NM	*t*-value	*P*-value	AM	NM	*t*-value	*P*-value
NERICA4-Upl	4.763	4.670	2.280	0.085	4.748	4.748	0.000	1.000
WAB56-104-Upl	4.801	4.758	1.028	0.362	4.754	4.754	0.000	1.000
CG14-Upl	**4.691**	**4.660**	**4.533**	**0.011**	4.718	4.713	0.999	0.374
IR64-Irr	4.804	4.804	0.000	1.000	4.939	4.939	0.000	1.000
Sahel202-Irr	4.787	4.768	2.079	0.106	4.903	4.903	0.000	1.000
Sahel108-Irr	4.679	4.685	-0.707	0.519	4.756	4.739	0.903	0.417
NERICA-L-19-Rll	4.817	4.795	0.785	0.477	4.900	4.900	0.000	1.000

TOG5681-Rll	4.663	4.663	0.000	1.000	4.685	4.685	0.000	1.000
Grain filling duration (GFD)								
	First year trial				Second year trial			
Variety	AM	NM	*t*-value	*P*-value	AM	NM	*t*-value	*P*-value
NERICA4-Upl	3.740	3.705	0.431	0.689	3.719	3.719	0.000	1.000
WAB56-104-Upl	3.883	3.768	1.108	0.330	3.752	3.752	0.000	1.000
CG14-Upl	3.571	3.406	1.758	0.154	3.636	3.712	-1.291	0.266
IR64-Irr	3.702	3.792	-1.465	0.216	3.721	3.721	0.000	1.000
Sahel202-Irr	3.585	3.499	0.693	0.527	3.769	3.769	0.000	1.000
Sahel108-Irr	3.340	3.465	-1.971	0.120	3.720	3.711	0.132	0.902
NERICA-L-19-Rll	3.494	3.524	-0.455	0.673	3.744	3.744	0.000	1.000
TOG5681-Rll	3.608	3.610	-0.030	0.977	3.523	3.523	0.000	1.000
Fertility								
	First year trial				Second year trial			
Variety	AM	NM	*t*-value	*P*-value	AM	NM	*t*-value	*P*-value
NERICA4-Upl	4.495	3.300	2.665	0.056	**4.592**	**4.392**	**5.378**	**0.006**
WAB56-104-Upl	4.416	4.170	2.360	0.078	4.501	4.431	1.459	0.218
CG14-Upl	**4.450**	**4.303**	**3.253**	**0.031**	4.579	4.562	1.894	0.131
IR64-Irr	4.303	4.175	0.916	0.412	4.630	4.583	1.978	0.119
Sahel202-Irr	4.228	4.072	1.278	0.270	4.618	4.571	1.794	0.147
Sahel108-Irr	4.429	4.239	1.847	0.138	**4.626**	**4.600**	**3.805**	**0.019**
NERICA-L-19-Rll	4.423	4.267	2.334	0.080	4.550	4.481	1.320	0.257
TOG5681-Rll	4.321	4.302	0.106	0.921	4.562	4.594	-0.821	0.458
