# Supplementary material for: Field Trials Reveal Ecotype-Specific Responses to Mycorrhizal Inoculation in Rice
Source: PLoS One. 2016 Dec 1;11(12):e0167014. doi: 10.1371/journal.pone.0167014 (PMC5132163; doi:10.1371/journal.pone.0167014)
Supplement: S4 Table — AMF inoculation (inoculated and non-inoculated), ecotype (upland, irrigated and rainfed lowland) and year (1st and 2nd-year trial). (PDF) [file pone.0167014.s006.pdf]

S4 Table. ANOVA for the ln (x +10) transformed values of agronomic traits in rice plants at ecotype level. AMF inoculation (inoculated and non-inoculated), ecotype (upland, irrigated and rainfed lowland) and year (1st and 2nd-year trial).

Yield					
Source of variation	df	*SS*	*MS*	*F*	*P*
AMF inoculation (AMF n = 2)	1	0.322	0.322	1.801	0.183
Ecotype (Eco n = 3)	2	7.500	3.750	20.985	< 0.0001
Year (Yr n = 2)	1	0.529	0.529	2.962	0.089
AMF x Eco	2	2.391	1.196	6.690	0.002
AMF x Yr	1	0.185	0.185	1.033	0.312
Eco x Yr	2	3.244	1.622	9.076	0.000
AMF x Eco x Yr	2	0.696	0.348	1.948	0.149
Error	84				
Biomass					
Source of variation	df	*SS*	*MS*	*F*	*P*
AMF inoculation (AMF n = 2)	1	0.001	0.001	0.009	0.924
Ecotype (Eco n = 3)	2	5.722	2.861	22.091	< 0.0001
Year (Yr n = 2)	1	2.164	2.164	16.705	< 0.0001
AMF x Eco	2	0.236	0.118	0.909	0.407
AMF x Yr	1	0.079	0.079	0.609	0.437
Eco x Yr	2	0.288	0.144	1.113	0.333
AMF x Eco x Yr	2	0.153	0.076	0.590	0.557
Error	84				
Harvest index (HI)					
Source of variation	df	*SS*	*MS*	*F*	*P*
AMF inoculation (AMF n = 2)	1	0.601	0.601	8.536	0.004
Ecotype (Eco n = 3)	2	0.222	0.111	1.576	0.213
Year (Yr n = 2)	1	0.864	0.864	12.259	0.001
AMF x Eco	2	0.794	0.397	5.632	0.005
AMF x Yr	1	0.131	0.131	1.853	0.177
Eco x Yr	2	1.279	0.640	9.080	0.000
AMF x Eco x Yr	2	0.072	0.036	0.514	0.600
Error	84				
1000 grains weight (1000GWT)					
Source of variation	df	*SS*	*MS*	*F*	*P*
AMF inoculation (AMF n = 2)	1	0.004	0.004	0.113	0.738
Ecotype (Eco n = 3)	2	0.347	0.173	4.435	0.015
Year (Yr n = 2)	1	0.062	0.062	1.579	0.212
AMF x Eco	2	0.060	0.030	0.762	0.470
AMF x Yr	1	0.002	0.002	0.041	0.840

Eco x Yr	2	0.057	0.029	0.730	0.485
AMF x Eco x Yr	2	0.096	0.048	1.234	0.296
Error	84				
		Height			
Source of variation	df	*SS*	*MS*	*F*	*P*
AMF inoculation (AMF n = 2)	1	0.001	0.001	0.073	0.788
Ecotype (Eco n = 3)	2	0.556	0.278	36.745	< 0.0001
Year (Yr n = 2)	1	0.011	0.011	1.404	0.239
AMF x Eco	2	0.010	0.005	0.689	0.505
AMF x Yr	1	0.000	0.000	0.009	0.927
Eco x Yr	2	0.014	0.007	0.918	0.403
AMF x Eco x Yr	2	0.019	0.009	1.230	0.298
Error	84				
		Tillers			
Source of variation	df	*SS*	*MS*	*F*	*P*
AMF inoculation (AMF n = 2)	1	0.000	0.000	0.001	0.979
Ecotype (Eco n = 3)	2	3.661	1.831	22.392	< 0.0001
Year (Yr n = 2)	1	4.333	4.333	52.998	< 0.0001
AMF x Eco	2	0.135	0.068	0.828	0.441
AMF x Yr	1	0.266	0.266	3.256	0.075
Eco x Yr	2	0.072	0.036	0.439	0.646
AMF x Eco x Yr	2	0.082	0.041	0.501	0.608
Error	84				
		Heading			
Source of variation	df	*SS*	*MS*	*F*	*P*
AMF inoculation (AMF n = 2)	1	0.005	0.005	0.777	0.381
Ecotype (Eco n = 3)	2	0.291	0.145	20.817	< 0.0001
Year (Yr n = 2)	1	0.045	0.045	6.384	0.013
AMF x Eco	2	0.001	0.000	0.043	0.958
AMF x Yr	1	0.002	0.002	0.223	0.638
Eco x Yr	2	0.021	0.010	1.483	0.233
AMF x Eco x Yr	2	0.000	0.000	0.002	0.998
Error	84				
		Maturity			
Source of variation	df	*SS*	*MS*	*F*	*P*
AMF inoculation (AMF n = 2)	1	0.004	0.004	0.755	0.387
Ecotype (Eco n = 3)	2	0.109	0.054	10.089	0.000
Year (Yr n = 2)	1	0.085	0.085	15.705	0.000
AMF x Eco	2	0.003	0.002	0.295	0.745
AMF x Yr	1	0.003	0.003	0.490	0.486
Eco x Yr	2	0.039	0.020	3.662	0.030
AMF x Eco x Yr	2	0.004	0.002	0.345	0.709

Source of variation	df	*SS*	*MS*	*F*	*P*
Error	84				
Grain filling duration (GFD)					
Source of variation	df	*SS*	*MS*	*F*	*P*
AMF inoculation (AMF n = 2)	1	0.000	0.000	0.020	0.888
Ecotype (Eco n = 3)	2	0.147	0.074	4.161	0.019
Year (Yr n = 2)	1	0.204	0.204	11.541	0.001
AMF x Eco	2	0.017	0.009	0.493	0.613
AMF x Yr	1	0.003	0.003	0.172	0.680
Eco x Yr	2	0.086	0.043	2.420	0.095
AMF x Eco x Yr	2	0.039	0.019	1.096	0.339
Error	84				
Fertility					
Source of variation	df	*SS*	*MS*	*F*	*P*
AMF inoculation (AMF n = 2)	1	0.494	0.494	13.901	0.000
Ecotype (Eco n = 3)	2	0.101	0.051	1.428	0.246
Year (Yr n = 2)	1	1.978	1.978	55.705	< 0.0001
AMF x Eco	2	0.244	0.122	3.435	0.037
AMF x Yr	1	0.209	0.209	5.878	0.017
Eco x Yr	2	0.076	0.038	1.077	0.345
AMF x Eco x Yr	2	0.116	0.058	1.630	0.202
Error	84				
