# Supplementary material for: Field Trials Reveal Ecotype-Specific Responses to Mycorrhizal Inoculation in Rice
Source: PLoS One. 2016 Dec 1;11(12):e0167014. doi: 10.1371/journal.pone.0167014 (PMC5132163; doi:10.1371/journal.pone.0167014)
Supplement: S5 Table — AM: inoculated with AMF and NM: non-inoculated. (PDF) [file pone.0167014.s007.pdf]

S5 Table. Student's t-test for the ln (x +10) transformed values of agronomic traits in rice plants at ecotype level. AM: inoculated with AMF and NM: non-inoculated.

Yield								
	First year trial				Second year trial			
Ecotype	AM	NM	*t*-value	*P*-value	AM	NM	*t*-value	*P*-value
Upland	**8.041**	**7.213**	**2.151**	**0.047**	**8.358**	**8.103**	**2.589**	**0.020**
Irrigated	8.441	8.490	-0.564	0.580	8.743	8.577	1.141	0.271
Rainfed lowland	8.460	8.618	-0.874	0.403	**8.026**	**8.362**	**-2.317**	**0.043**
Biomass								
	First year trial				Second year trial			
Ecotype	AM	NM	*t*-value	*P*-value	AM	NM	*t*-value	*P*-value
Upland	8.949	8.759	0.853	0.406	9.232	9.344	-0.672	0.511
Irrigated	9.523	9.461	0.569	0.577	9.737	9.609	0.780	0.447
Rainfed lowland	9.364	9.462	-0.681	0.511	9.610	9.821	-0.847	0.417
Harvest index (HI)								
	First year trial				Second year trial			
Ecotype	AM	NM	*t*-value	*P*-value	AM	NM	*t*-value	*P*-value
Upland	**4.059**	**3.509**	**3.440**	**0.003**	**3.947**	**3.671**	**2.884**	**0.011**
Irrigated	3.938	3.877	1.073	0.299	3.867	3.827	0.284	0.780
Rainfed lowland	4.082	3.983	1.049	0.319	3.460	3.517	-0.250	0.807
1000 grains weight (1000GWT)								
	First year trial				Second year trial			
Ecotype	AM	NM	*t*-value	*P*-value	AM	NM	*t*-value	*P*-value
Upland	3.672	3.530	0.837	0.415	3.485	3.478	0.138	0.892
Irrigated	3.498	3.432	1.025	0.321	3.426	3.451	-0.338	0.740
Rainfed lowland	3.536	3.678	-2.105	0.062	3.615	3.581	0.447	0.664
Height								
	First year trial				Second year trial			
Ecotype	AM	NM	*t*-value	*P*-value	AM	NM	*t*-value	*P*-value
Upland	4.670	4.674	-0.095	0.926	4.643	4.677	-0.895	0.384
Irrigated	4.475	4.513	-1.154	0.265	4.548	4.511	1.193	0.250
Rainfed lowland	4.516	4.454	0.880	0.399	4.529	4.522	0.161	0.876
Tillers								
	First year trial				Second year trial			
Ecotype	AM	NM	*t*-value	*P*-value	AM	NM	*t*-value	*P*-value
Upland	5.294	5.399	-0.499	0.625	5.949	5.679	1.714	0.106
Irrigated	5.756	5.798	-0.453	0.656	6.178	6.091	0.846	0.410
Rainfed lowland	5.692	5.872	-1.570	0.147	6.235	6.275	-0.548	0.595
Heading								
	First year trial				Second year trial			
Ecotype	AM	NM	*t*-value	*P*-value	AM	NM	*t*-value	*P*-value
Upland	4.428	4.398	1.227	0.237	4.428	4.413	1.113	0.282
Irrigated	4.515	4.493	0.968	0.348	4.582	4.575	0.119	0.907
Rainfed lowland	4.490	4.471	0.255	0.804	4.530	4.530	0.000	1.000
Maturity								

	First year trial				Second year trial			
Ecotype	AM	NM	*t*-value	*P*-value	AM	NM	*t*-value	*P*-value
Upland	4.752	4.696	1.996	0.063	4.740	4.738	0.203	0.842
Irrigated	4.757	4.752	0.170	0.867	4.866	4.860	0.135	0.894
Rainfed lowland	4.740	4.729	0.236	0.818	4.793	4.793	0.000	1.000
Grain filling duration (GFD)								
	First year trial				Second year trial			
Ecotype	AM	NM	*t*-value	*P*-value	AM	NM	*t*-value	*P*-value
Upland	3.731	3.626	1.227	0.238	3.702	3.728	-0.737	0.472
Irrigated	3.542	3.585	-0.497	0.626	3.736	3.733	0.109	0.914
Rainfed lowland	3.551	3.567	-0.310	0.763	3.634	3.634	0.000	1.000
Fertility								
	First year trial				Second year trial			
Ecotype	AM	NM	*t*-value	*P*-value	AM	NM	*t*-value	*P*-value
Upland	**4.454**	**3.977**	**2.704**	**0.016**	**4.557**	**4.463**	**2.786**	**0.013**
Irrigated	**4.320**	**4.162**	**2.193**	**0.043**	**4.625**	**4.584**	**3.536**	**0.003**
Rainfed lowland	4.372	4.285	1.003	0.340	4.556	4.538	0.476	0.644
